# Patient-Friendly Test Results and Patient-Initiated Messaging Among Adult Outpatients

**DOI:** 10.1001/jamanetworkopen.2025.43879

**Published:** 2025-11-17

**Authors:** Bryan D. Steitz, Andrew Guide, Kelsey Rodriguez, Sunil Kripalani, Chetan V. Aher, Kaylin S. Craig, Romney M. Humphries, Amanda S. Mixon, Christianne L. Roumie, Adam Wright, S. Trent Rosenbloom

**Affiliations:** 1 Department of Biomedical Informatics, Vanderbilt University Medical Center, Nashville, Tennessee; 2 Department of Biostatistics, Vanderbilt University Medical Center, Nashville, Tennessee; 3 Center for Health Services Research, Vanderbilt University Medical Center, Nashville, Tennessee; 4 Department of Medicine, Vanderbilt University Medical Center, Nashville, Tennessee; 5 Department of Health Policy, Vanderbilt University Medical Center, Nashville, Tennessee; 6 Department of Surgery, Vanderbilt University Medical Center, Nashville, Tennessee; 7 Division of General Internal Medicine and Public Health, Vanderbilt University Medical Center, Nashville, Tennessee; 8 Department of Pathology, Microbiology and Immunology, Vanderbilt University Medical Center, Nashville, Tennessee; 9 Center for Quality Aging, Vanderbilt University Medical Center, Nashville, Tennessee; 10 Geriatric Research Education and Clinical Center, Veterans Affairs (VA) Tennessee Valley Healthcare System, Nashville; 11 Center for Clinical Quality and Implementation Research, Vanderbilt University Medical Center, Nashville, Tennessee; 12 Veteran Administration Tennessee Valley VA Health Care System Geriatric Research Education Clinical Center, Veterans’ Wellbeing Through Innovation, Systems Science and Experience in Learning Health Systems, Nashville, Tennessee; 13 Department of Pediatrics, Vanderbilt University Medical Center, Nashville, Tennessee

## Abstract

**Importance:**

Patients have access to test results but often struggle with interpretation due to limited context. Helping patients understand their results is essential for engagement and may reduce unnecessary messages requesting explanation.

**Objective:**

To test the hypothesis that releasing test results in a patient-friendly educational format would reduce rates of patient-initiated messaging.

**Design, Setting, and Participants:**

This quality improvement study using an interrupted time series design with preintervention and postintervention evaluation was performed at a single academic medical center that implemented patient-friendly presentation of test results through a patient portal for results with the highest message volumes, including basic metabolic panel, comprehensive metabolic panel, complete blood cell count, thyroid panel or thyrotropin level, urine microalbumin tests, and selected microbial, antibody, and polymerase chain reaction (PCR) tests. Participants included adults who received outpatient test results via a patient portal from January 1 to December 31, 2024.

**Exposures:**

Educational format messaging.

**Main Outcomes and Measures:**

The primary outcome was the proportion of results reviewed by patients followed by a patient-initiated message within 24 hours of result release. An interrupted time series was performed to estimate the association of releasing results with the new educational format with messaging. Fitted models were stratified by whether results were ordered in primary care or flagged as abnormal.

**Results:**

A total of 829 902 results were reviewed by 205 139 patients (mean [SD] age, 51.0 [17.8] years; 130 284 [63.5%] female). After introducing educational messaging, nonsignificant decreases in weekly messages (mean [SD], 2769.2 [451.7] vs 2754.0 [529.6]; *P* = .92) and the proportion of results with a patient-initiated message within 24 hours (87 038 of 497 651 [17.5%] vs 57 585 of 332 251 [17.3%]; *P* = .22) were observed. When stratified by tests ordered through primary care, a statistically significant decrease was observed in messaging (average marginal effect, −0.8% [95% CI, −1.2% to −0.5%]; *P* < .001). Messaging was increased for microbial, antibody, and PCR tests ordered outside primary care (average marginal effect, 2.1% [95% CI, 1.0% to 3.1%]; *P* < .001).

**Conclusions and Relevance:**

In this quality improvement study in which test results were released in a patient-friendly educational format, no clinically meaningful change in patient messaging was observed. As organizations navigate information transparency and growing message volumes, it is essential to develop, implement, and study approaches to support information needs without impacting clinical workflow.

## Key Points

**Question:**

Among adult patients, is the release of test results via patient portal in a patient-friendly educational format associated with a decrease in patient-initiated messaging?

**Findings:**

In this quality improvement study including 205 139 patients who reviewed 829 902 test results during 2024, patient-initiated messaging decreased by 15 messages per week after the introduction of a patient-friendly educational format for test results. A statistically significant decrease of 0.8% in messages sent after reviewing results for tests ordered in primary care was observed.

**Meaning:**

These findings suggest that generalized patient-friendly educational materials may support patients reviewing their results but may not promote significant changes in patient messaging behavior.

## Teaser

This quality improvement study assesses whether patient-friendly educational information could reduce the weekly rates and proportions of patient-initiated messaging after review of test results via an online patient portal.

## Introduction

The 21st Century Cures Act requires that patients have immediate electronic access to their test results on request.^1^ Many health care organizations comply with this law by releasing all results to the patient portal as soon as they become available in the electronic health record (EHR). Historically, patients have had access to test results in the patient portal, but many results were released after a delay that was designed to allow clinicians time to review and follow up on sensitive findings when necessary.^2^ Since the Cures Act Information Blocking Provisions went into effect in 2021, patients view more than 45% of test results before their ordering clinician.^3^ As a result, patient-initiated messages to their clinical teams have doubled.^4^ Most patient portal users are satisfied receiving test results online, but more than 55% report seeking additional information to understand or contextualize their results.^5,6^

Transparent access to test results offers opportunities for patient engagement and to strengthen patient-clinician relationships.^7,8,9^ However, simply providing access to test results is insufficient, as many patients struggle to interpret their results.^7,10,11^ This can be due to the complexity of medical data, limited health literacy, lack of familiarity with medical terminology, and limited context about the implications for one’s health.^12,13,14,15^ These challenges are especially pronounced among individuals with lower educational attainment or limited health literacy, potentially exacerbating health disparities.^12,14,16^ To address this gap, patient portals can offer educational materials, but these materials may not be available at the time of result review and may lack clear explanations and actionable guidance.^14,17^ In response, patients often turn to their clinical team via secure messaging to seek clarification, contributing to undue patient worry and adding to clinical work.^5,6,7,18^

As organizations balance benefits of improved information availability alongside a 2-fold increase in patient-initiated messages since 2019,^19,20^ there is an opportunity for proactive strategies that support patients in reviewing and interpreting their test results. Providing patient-friendly educational materials can help patients interpret their results, support informed decision-making, and improve clinical work and workflow by reducing excess communication. In August 2024, Vanderbilt University Medical Center (VUMC) implemented curated patient educational materials—including a patient-friendly description and basic interpretation of the test—at the point of result review for common tests previously associated with the highest message volumes. In this study, we tested the hypothesis that this patient-friendly educational format would reduce the weekly rates and the proportion of patient-initiated messaging following result review.

## Methods

### Study Setting

We conducted this study at VUMC, a large academic medical center in Nashville, Tennessee. Patients at VUMC can review their test results and message their clinical teams through My Health at Vanderbilt (MHAV), an online patient portal (MyChart-based patient portal; Epic Systems Corporation).^2^ The VUMC Institutional Review Board approved the study procedures and granted a waiver of informed consent because the research was conducted on data previously collected during routine patient care and was deemed no more than minimal risk. We followed the Standards for Quality Improvement Reporting Excellence (SQUIRE) and Reporting of Studies Conducted Using Observational Routinely Collected Health Data (RECORD) reporting guidelines.^21^

### Intervention and Time Periods

In this quality improvement study using an interrupted time series design, we evaluated the impact of the new educational results format integrated into the patient portal interface for select results released to adult patients. The intervention was deployed on August 7, 2024, and the laboratory tests for intervention included those associated with the highest message volumes. These tests included a basic metabolic panel, a comprehensive metabolic panel (CMP) including liver function tests (LFTs), complete blood cell count (CBC), a thyroid panel or thyrotropin tests, urine microalbumin tests, and selected microbial, antibody, and polymerase chain reaction (PCR) tests. The new patient-friendly results format was developed by health literacy experts within VUMC’s Department of Patient Education in collaboration with experts in internal medicine, laboratory medicine, and clinical informatics. A complete list of tests included within each category and their accompanying educational text is available in eTable 1 in Supplement 1. Educational content was presented after the result and included a description of the test, basic interpretation reinforced with graphic description of the result and reference range, and a note indicating that a member of the clinical team would follow up if results were of concern for the patient or required further action.

We studied patient messaging behavior after these tests were ordered during ambulatory outpatient encounters, and results were available to patients through MHAV. During the study, all results were released to MHAV as soon as they became available in the EHR. We extracted study data from the online portal’s reporting database supporting the clinical information system (Epic Clarity; Epic Systems Corporation) for the period between January 1, 2024, and December 31, 2024, divided before and after the intervention release on August 7.

### Outcome Measures

The individual test result was the unit of analysis. The primary outcome was patient initiation of a new message within 24 hours of result availability, which was recorded as a binary variable. This was measured as the rate of weekly messaging and the proportion of reviewed tests with a patient-initiated message. Test result details include the time of result release, time of patient review, time of clinician review, whether the result was ordered in a primary care setting, and whether the result was flagged as abnormal. We indicated that the test was ordered in a primary care setting if the ordering clinician delivered care in a clinic with a specialty of student health, primary care, or internal medicine.

### Covariates

For each test result, we collected patient sociodemographic variables. Patient sociodemographic variables included age groups (18-34, 35-49, 50-64, 65-84, and ≥85 years), legal sex, race, ethnicity, preferred language, and insurance as documented in the EHR. Self-reported race categories included American Indian or Alaska Native, Asian, Black or African American, Middle Eastern or North African, Native Hawaiian or Other Pacific Islander, White, and other (including none of these, other, prefer not to answer, and unable to provide). Self-reported ethnicity variables included Hispanic or Latino, non-Hispanic or non-Latino, and other (including unknown, prefer not to answer, or unable to provide). Racial and ethnic data were included because racial and ethnic disparities have been associated with lower rates of portal use in prior studies.^22^ During the study, MHAV was available in English and Spanish, so we report preferred language as English, Spanish, or other. We classified primary insurance as commercial (including commercial, agency, exchange, or managed care), Medicaid, Medicare, uninsured, and other (including workers’ compensation or other nonclassified payers).

### Statistical Analysis

We calculated descriptive statistics stratified by whether the result was viewed within the portal. We compared demographic characteristics of patients who reviewed their test results, using a χ^2^ test for categorical variables and a 1-way analysis of variance for continuous variables. We compared the proportions of tests with a patient-initiated message within 24 hours of release using 2-sample *z* tests for proportions. We tested statistically significant differences in weekly result review and messaging between test type categories using 2-tailed *t* tests with unequal variances.

We conducted interrupted time series analyses to estimate the association of the new educational format with patient-initiated messaging in the time periods before and after August 7, 2024, when the patient-friendly laboratory reports were first released. We used multivariable segmented logistic regression models to assess the change in proportion of reviewed results with a patient-initiated message. We also fit multivariable segmented logistic models, stratified by whether the test was ordered in primary care and whether the result was abnormal. For each model, we computed SEs, clustered by patient, to account for patients who received multiple results. We transformed odds ratios to average marginal effects (AMEs), which represent the average change in the probability of a patient sending a message after reviewing results with the new educational format. We report AMEs with 95% CIs. All models controlled for patient and result characteristics including the aforementioned covariates and whether the result was reviewed first by the patient or clinician.

Statistical significance was set at α = .05. Analyses were performed using R, version 4.4.3 (R Foundation for Statistical Computing).

## Results

During the study, we observed 228 783 patients who received 1 012 972 results. Of these, 205 139 patients (89.7%) reviewed 829 902 results (81.9%). Among patients who reviewed results, 130 284 (63.5%) were female, 74 852 (36.5%) were male, and 3 (0.01%) were of unknown sex, with a mean (SD) age of 51.0 (17.8) years at the time of their first ordered test. In terms of race, 817 (0.4%) were American Indian or Alaska Native, 5535 (2.7%) were Asian, 21 838 (10.6%) were Black or African American, 789 (0.4%) were Middle Eastern or North African, 202 (0.1%) were Native Hawaiian or Other Pacific Islander, 162 216 (79.1%) were White, and 13 742 (6.7%) were of other or unknown race. In addition, 10 386 patients (5.1%) were Hispanic, 172 633 (84.2%) were non-Hispanic, and 22 120 (10.8%) were of other or unknown ethnicity. Most patients spoke English speaking (201 478 [98.2%]), compared with 2059 (1.0%) who spoke Spanish and 1602 (0.8%) who spoke another language. Most viewed results (531 823 [64.1%]) were flagged as abnormal. There were 575 582 results (69.4%) reviewed by patients before their clinicians. Of the reviewed results, patients initiated a message for 144 623 (17.4%) within 24 hours of result availability (Table 1). Conversely, patients initiated a message within 24 hours in 12 433 unreviewed results (6.8%). Patient demographic characteristics and the characteristics of test results reviewed by patients stratified by test category are available in eTable 2 in Supplement 1.

**Table 1.** Characteristics of Patients Receiving Test Results via the Patient Portal

Characteristic	Test results, No. (%)			*P* value
	Never viewed (n = 183 070)	Viewed (n = 829 902)	All (N = 1 012 972)	
Age group, y				
18-34	24 311 (13.3)	152 472 (18.4)	176 783 (17.5)	<.001
35-49	28 189 (15.4)	181 815 (21.9)	210 004 (20.7)	
50-64	54 124 (29.6)	243 437 (29.3)	297 561 (29.4)	
65-84	70 198 (38.3)	239 699 (28.9)	309 897 (30.6)	
≥85	6248 (3.4)	12 479 (1.5)	18 727 (1.8)	
Sex				
Female	95 903 (52.4)	495 987 (59.8)	591 890 (58.4)	<.001
Male	87 167 (47.6)	333 907 (40.2)	421 074 (41.6)	
Unknown^a^	0	8 (0.001)	8 (0.001)	
Ethnicity				
Hispanic or Latino	10 634 (5.8)	38 468 (4.6)	49 102 (4.8)	<.001
Non-Hispanic or non-Latino	152 278 (83.2)	717 865 (86.5)	870 143 (85.9)	
Other or unknown^b^	20 158 (11.0)	73 569 (8.9)	93 727 (9.3)	
Race				
American Indian or Alaska Native	945 (0.5)	3646 (0.4)	4591 (0.5)	<.001
Asian	2712 (1.5)	20 143 (2.4)	22 855 (2.3)	
Black or African American	39 975 (21.8)	89 226 (10.8)	129 201 (12.8)	
Middle Eastern or North African	721 (0.4)	3279 (0.4)	4000 (0.4)	
Native Hawaiian or Pacific Islander	278 (0.2)	1050 (0.1)	1328 (0.1)	
White	126 310 (69.0)	666 969 (80.4)	793 279 (78.3)	
Other or unknown^c^	12 129 (6.6)	45 589 (5.5)	57 718 (5.7)	
Preferred language				
English	174 924 (95.6)	816 708 (98.4)	991 632 (97.9)	<.001
Spanish	2969 (1.6)	6144 (0.7)	9113 (0.9)	
Other^d^	5177 (2.8)	7050 (0.8)	12 227 (1.2)	
Insurance				
Commercial	66 676 (36.4)	494 957 (59.6)	561 633 (55.4)	<.001
Medicaid	6881 (3.8)	26 871 (3.2)	33 752 (3.3)	
Medicare	76 134 (41.6)	276 697 (33.3)	352 831 (34.8)	
Uninsured	28 725 (15.7)	10 472 (1.3)	39 197 (3.9)	
Other^e^	4654 (2.5)	20 905 (2.5)	25 559 (2.5)	
Time enrolled in portal, y				
Mean (SD)	2.2 (2.9)	3.4 (2.4)	3.2 (2.5)	<.001
Median (IQR)	2.2 (0.04-4.6)	3.4 (1.2-6.0)	3.2 (1.0-5.9)	
Order specialty				
Primary care	34 914 (19.1)	161 108 (19.4)	196 022 (19.4)	<.001
Other	148 156 (80.9)	668 794 (80.6)	816 950 (80.6)	
Test findings				
Abnormal	124 767 (68.2)	531 823 (64.1)	656 590 (64.8)	<.001
Normal	58 303 (31.8)	298 079 (35.9)	356 382 (35.2)	
Patient-initiated message within 24 h				
No	170 637 (93.2)	685 279 (82.6)	855 916 (84.5)	<.001
Yes	12 433 (6.8)	144 623 (17.4)	157 056 (15.5)	

^a^ Unknown as indicated in the electronic health record (EHR).

^b^ Includes unknown, prefer not to answer, or unable to provide as indicated in the EHR.

^c^ Includes none of these, other, prefer not to answer, and unable to provide as indicated in the EHR.

^d^ Includes, for example, Arabic, Japanese, and Vietnamese.

^e^ Includes workers’ compensation and nonclassified payers.

### Primary Outcome: Patient-Initiated Messaging

The weekly message volume and the proportion of results with a subsequent message remained stable following the introduction of the new results format (Table 2). Overall, we observed a small, nonsignificant decrease in weekly message volume (mean [SD], 2769.2 [451.7] vs 2754.0 [529.6]; *P* = .92) The proportion of viewed results in which a message was sent within 24 hours of receiving the result remained stable between the preintervention and postintervention periods (87 038 of 497 651 [17.5%] vs 57 585 of 332 251 [17.3%]; *P* = .22), representing an average decrease of 15 messages per week. When stratified by test type, none of the test types demonstrated a statistically significant change between the preintervention and postintervention periods.

**Table 2.** Absolute Change in Weekly Message Volume by Result Type^a^

Test category	No. of tests		Mean (SD)				*P* value
			No. of weekly messages		Tests with message in 24 h, %		
	Preintervention	Postintervention	Preintervention	Postintervention	Preintervention	Postintervention	
BMP	35 304	23 925	219.1 (41.7)	229.0 (44.4)	19.4 (1.6)	20.0 (1.3)	.43
CMP and LFTs	170 739	114 115	944.7 (152.0)	929.0 (176.8)	17.5 (1.2)	17.0 (0.7)	.75
CBC	188 089	126 153	1048.9 (165.6)	1043.7 (191.9)	17.6 (1.0)	17.3 (0.4)	.92
Microbial, antibody, and PCR	18 188	12 423	147.0 (28.2)	162.2 (36.5)	25.3 (3.1)	27.1 (3.5)	.12
Thyroid panel and thyrotropin levels	72 539	47 741	350.5 (68.0)	336.0 (76.8)	15.3 (1.2)	14.6 (1.2)	.49
Urine microalbumin	12 792	7894	59.0 (15.1)	55.1 (14.8)	14.4 (2.2)	14.3 (2.2)	.37
All tests	497 651	332 251	2769.2 (451.7)	2754.0 (529.6)	17.5 (0.9)	17.3 (0.6)	.92

Abbreviations: BMP, basic metabolic panel; CBC, complete blood cell count; CMP, comprehensive metabolic panel; LFT, liver function test; PCR, polymerase chain reaction.

^a^ There were 31 weeks in the preintervention period and 20 weeks in the postintervention period. Intervention deployment occurred during week 32, which was excluded from analysis.

**Figure 1.**
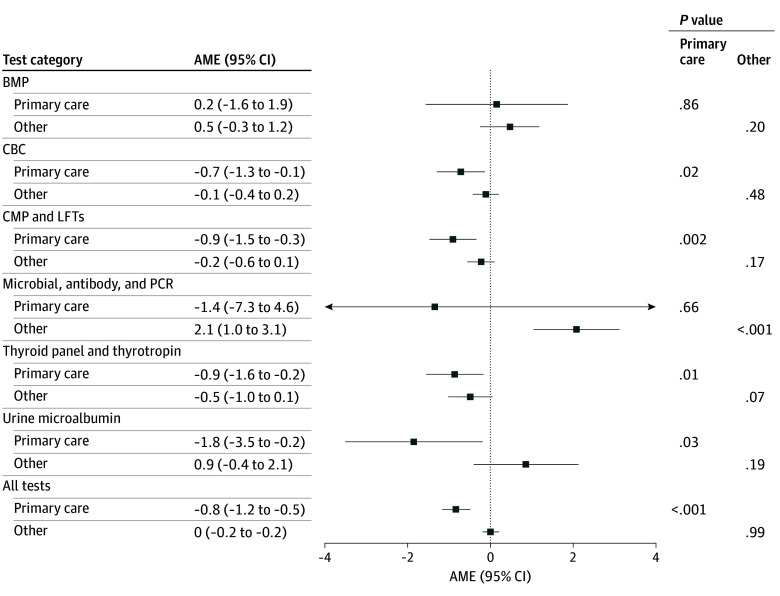
Average Marginal Effects (AMEs) of Educational Materials on Patient-Initiated Messaging Within 24 Hours of Result Availability Stratified by Order Setting A positive AME represents an increase in patient-initiated messaging after releasing results in the new educational format. Models adjust for age group, sex, ethnicity, race, preferred language, insurance type, time enrolled in the portal at result release, and whether the patient reviewed the result before their clinician. BMP indicates basic metabolic panel; CBC, complete blood cell count; CMP, comprehensive metabolic panel; LFT, liver function test; and PCR, polymerase chain reaction.

When results were stratified by care setting, we observed a statistically significant decrease in the proportion of messages after reviewing tests ordered in primary care (AME, −0.8% [95% CI, −1.2% to −0.5%]; *P* < .001) overall and by certain test types (Figure 1). There was a statistically significant decrease in messaging after review of CBCs (AME, −0.7% [95% CI, −1.3% to −0.1%]; *P* = .02), CMP and LFTs (AME, −0.9% [95% CI, −1.5% to −0.3%]; *P* = .002), thyroid panels and thyrotropin levels (AME, −0.9% [95% CI, −1.6% to −0.2%]; *P* = .01), and urine microalbumin tests (AME, −1.8% [95% CI, −3.5% to −0.2%]; *P* = .03) ordered in primary care. We observed a statistically significant increase in messaging after review of microbial, antibody, and PCR tests that were ordered outside of primary care (AME, 2.1% [95% CI, 1.0%-3.1%]; *P* < .001); however, there was no change in messaging across all tests ordered in specialty care.

**Figure 2.**
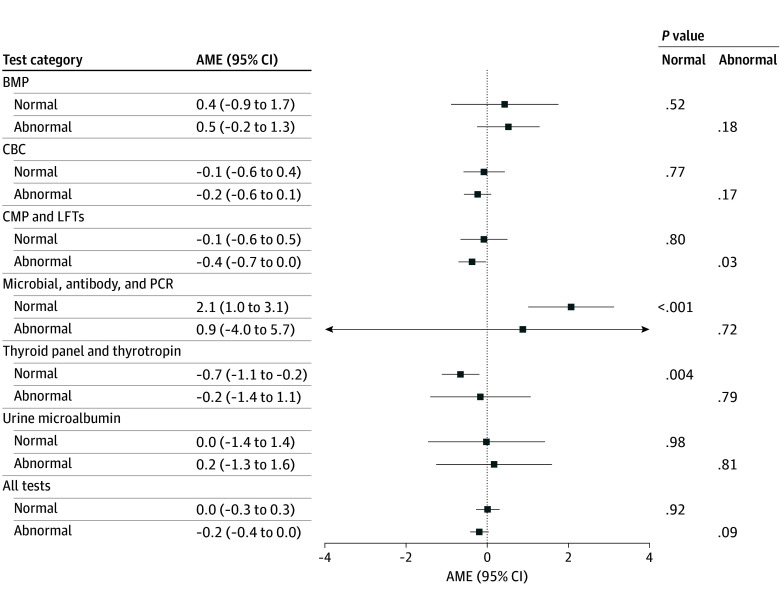
Average Marginal Effects (AMEs) of Educational Materials on Patient-Initiated Messaging Within 24 Hours of Result Availability Stratified by Abnormal Flagged Results A positive AME represents an increase in patient-initiated messaging after releasing results in the new educational format. Models adjust for age group, sex, ethnicity, race, preferred language, insurance type, time enrolled in the portal at result release, and whether the patient reviewed the result before their clinician. BMP indicates basic metabolic panel; CBC, complete blood cell count; CMP, comprehensive metabolic panel; LFT, liver function test; and PCR, polymerase chain reaction.

When stratified by normal vs abnormal results (Figure 2), we observed a slight but significant decrease in patient-initiated messaging for abnormal CMP and LFT results (AME, −0.4% [95% CI, −0.7% to 0.03%]; *P* = .03) and normal thyroid panel and thyrotropin test results (AME, −0.7% [95% CI, −1.1% to −0.2%]; *P* = .004). Conversely, we observed a statistically significant increase for normal microbial, antibody, and PCR tests (AME, 2.1% [95% CI, 1.0%-3.1%]; *P* < .001).

## Discussion

This study of more than 200 000 patients and 1 million test results found that sharing patient-friendly educational materials with released test results had limited associations with patient-initiated messaging. Prior to the introduction of educational materials, we observed that patients initiated a message within 24 hours of release for 17.5% of results, compared with 17.3% of results when educational materials were available, representing an average decrease of 15 messages per week. When we stratified by location of care, we observed a statistically significant 0.8 percentage-point decrease in messaging after including patient educational materials for those tests ordered in primary care. We did not find any statistically significant changes in messaging behavior for tests ordered outside of primary care or when the result was noted as either normal or abnormal. These findings indicate that while educational materials may have supported patients, they were not associated with a clinically meaningful decrease in messaging in the period following result review.

Prior work^5^ has demonstrated that most patients seek additional information after reviewing their results, often turning to online resources or contacting health care clinicians to satisfy information needs.^23,24^ However, we found that the provided patient-friendly educational materials were not associated with a clinically meaningful change in messaging behavior. Several factors may explain these findings. One possibility is that the educational materials did not fully address patient questions or concerns. Future work should explore whether more detailed or personalized educational content can reduce patient messages. Some patients may also reach out regardless of educational resources, particularly when confronted with new or uncertain findings, because clinicians remain a trusted source of advice.^25,26,27^ Finally, messaging frequency is an imperfect measure of patient understanding of their result, as patients send messages for a variety of reasons. In addition, the phrasing in the educational text that discouraged patients from calling rather than messaging may have unintentionally led some patients to view secure messaging as a less burdensome alternative.

The increase in message volume following the immediate availability of test results has occurred alongside a broader trend of increased administrative workload.^19,28^ Health systems have introduced various strategies aimed at supporting patients while mitigating the flow of messages. For example, VUMC changed default notification settings that allow patients to indicate their preference for being alerted and opt in to receiving notifications for new results.^3^ This change in notification settings was intended to reduce negative emotional response and messages from patients who prefer clinician follow-up and interpretation before reviewing their results. Additionally, prior research has demonstrated that precounseling patients about the purpose of a test, possible outcomes, and expectations for follow-up was associated with lesser odds of worry after result review.^5^ Precounseling at the time of test ordering is a promising strategy to improve patient experience and reduce messaging that merits further study.^29,30^

Although our educational materials were not associated with a reduction in patient messaging, they may still contribute to improved patient experience. Many patients report feeling more engaged in their care when they are given timely access to health information.^31,32^ This empowers them to formulate questions, seek clarification, and prepare for follow-up. Additional research should measure the impact of releasing test results with accompanying educational materials on patient experience and satisfaction. Future research should also evaluate how to refine educational content, including tailoring materials to patient context, incorporating patient input during design, and analyzing message content to identify information gaps. In addition, subgroup differences in messaging behaviors merit focused study, although our analysis was not designed for this purpose.

Educational materials were associated with a significant reduction in patient messages for tests ordered in primary care settings, both overall and for individual categories of tests, including CBCs, CMPs and LFTs, thyroid panel and thyrotropin levels, and urine microalbumin tests. We did not observe a change in messaging for tests ordered outside primary care. There are several possible explanations for this finding. Tests ordered in specialty contexts may involve complex clinical questions, nuanced diagnostic reasoning, or patient populations with more serious health conditions, all of which may prompt follow-up messaging. In contrast, tests in primary care are often ordered for routine monitoring by a clinician with whom there is a longitudinal relationship. These factors may contribute to patient ease of interpretation with basic educational materials.

When we stratified our analysis by whether test findings were reported as abnormal, we observed an association with significantly increased messaging for normal microbial, antibody, and PCR test results. This increase may have been due, in part, to the diversity of tests within this category that may not be adequately explained by a single description meant to encompass all results. It is also possible that the nonspecific patient educational materials related to this category introduced confusion or concern for patients who expected more definitive reassurance from a normal result. Even when test results are clinically unremarkable, patients may still experience uncertainty that prompts them to send a follow-up communication. Future research should analyze the content of these messages, which was beyond the scope of the present study, to help clarify underlying concerns that prompt a patient message and inform more targeted educational strategies.

### Limitations

This study has several limitations. First, we studied adult patients receiving test results in an ambulatory care setting at a single academic medical center that uses a specific online patient portal. The patient population enrolled in MHAV is disproportionally female, non-Hispanic White, and English speaking, which may limit the generalizability of our findings. Further, our analysis did not account for patient notification preferences. This omission may influence messaging behavior, although VUMC’s opt-in notification policy is not widely adopted and may also limit generalizability. Additional work should validate our findings across organizations and patient populations. Second, this interrupted time series is subject to unmeasured confounding factors that may have influenced messaging behavior independently of the introduction of educational materials. Third, although educational materials were developed with expert input, their content, tone, and presentation may not have optimally addressed patient needs. Future work should consider ways to refine educational materials, such as through codesign with patients or analysis of message content. Fourth, the phrasing of the educational material accompanying test results discouraged calling rather than messaging. While this wording was intended to broadly discourage unnecessary follow-up, it may have inadvertently led some patients to perceive secure messaging as a less burdensome alternative. Finally, we assessed changes in messaging without evaluating content, and patients may message for unrelated needs. Interestingly, we observed that 6.8% of unreviewed results had a patient-initiated message within 24 hours of result release, suggesting variation in message topic. Natural language processing to understand message content is an important area for future exploration.

## Conclusions

This quality improvement study using an interrupted time series design found that sharing patient-friendly educational materials with test results had limited association with reducing patient-initiated messaging. We observed a modest decrease in messaging for tests ordered in primary care, and a small increase in messages sent for microbial, antibody, and PCR tests. These findings suggest that generalized educational materials may have limited influence on patient messaging behavior and highlight the opportunity for more context-sensitive individualized approaches, especially when results are clinically complex or ambiguous. As patients access immediately available results, health systems must consider more nuanced strategies that support patient understanding while managing clinician workload. Future efforts should evaluate different strategies, including tailored educational content, delivered at both the time of test ordering and result review, or interventions that address patient expectations and preferences throughout the workflow, including test ordering, result release, and result review.

## Notes

Supplement 1. **eTable 1.** Test Results Within Each Category and the Accompanying Education Materials That Were Available in MHAV **eTable 2.** Characteristics of Patients Who Reviewed Test Results, Stratified by Test Type

Supplement 2.
Data Sharing Statement
