# Supplementary material for: Patient-Friendly Test Results and Patient-Initiated Messaging Among Adult Outpatients
Source: JAMA Netw Open. 2025 Nov 17;8(11):e2543879. doi: 10.1001/jamanetworkopen.2025.43879 (PMC12625389; doi:10.1001/jamanetworkopen.2025.43879)
Supplement: Supplement 1. — eTable 1. Test Results Within Each Category and the Accompanying Education Materials That Were Available in MHAV eTable 2. Characteristics of Patients Who Reviewed Test Results, Stratified by Test Type [file jamanetwopen-e2543879-s001.pdf]

Supplementary Online Content

Steitz BD, Guide A, Rodriguez K, et al. Patient-friendly test results and patient-initiated messaging among adult outpatients. *JAMA Netw Open.* 2025;8(11):e2543879. doi:10.1001/jamanetworkopen.2025.43879

eTable 1. Test Results Within Each Category and the Accompanying Education Materials That Were Available in MHAV

eTable 2. Characteristics of Patients Who Reviewed Test Results, Stratified by Test Type

This supplementary material has been provided by the authors to give readers additional information about their work.

© 2025 Steitz BD et al. *JAMA Network Open*.

eTable 1. Test Results Within Each Category and the Accompanying Education Materials That Were Available in MHAV

Test Result Category	Included Tests	Education Content
BMP	• BMP	These tell us about your body's metabolic health. This includes things like your:<br>• body's fluid balance<br>• sugar level<br>• sodium (salt) level<br>• potassium level.<br>If you see some numbers that are slightly outside of the normal range, you don't need to call us about it. We'll let you know if you need treatment.
CMP and LFTs	• CMP<br>• Hepatic Function Panel	These tell us how well your liver is working. It may also tell us about your metabolic health. If you see some numbers that are slightly outside of the normal range, you don't need to call us about it. We'll let you know if you need treatment.
CBC	• CBC<br>• CBC with Differential	This tells us about your blood. It includes:<br>• white blood cell count (to fight infection)<br>• hemoglobin (to carry oxygen)<br>• hematocrit (how much of your blood is made up of red blood cells)<br>• platelet count (to stop bleeding)<br>• and a few other things.<br>If you see some numbers that are slightly outside of the normal range, you don't need to call us about it. We'll let you know if you need treatment.
Thyroid Panel or TSH	• TSH	This tells us about your thyroid. If you have thyroid problems, it tells us if your treatment is working. If you see some numbers that are slightly outside of the normal range, you don't need to call us about it. We'll let you know if you need treatment.
Urine Microalbumin	• Urine Microalbumin Level<br>• Urine 24hr Microalbumin Level	This helps to tell us about your kidneys. We look at how much urine creatinine you have compared to your albumin. If you see numbers that are slightly outside of the normal range, you don't have to call us. We'll let you know if you need treatment.
Selected Microbial, Antibody, and PCR Tests	• Polymerase Chain Reaction Cytomegalovirus Quantitative<br>• Polymerase Chain Reaction Epstein-Barr Virus Quantitative<br>• Polymerase Chain Reaction BK Virus Quantitative<br>• Polymerase Chain Reaction Herpes Simplex Virus Type 1/2<br>• Polymerase Chain Reaction Hepatitis B Virus Quantitative<br>• Polymerase Chain Reaction Human Immunodeficiency Virus Type 1 RNA Quantitative<br>• Rapid Plasma Reagin Titer (Syphilis Test)<br>• Cerebrospinal Fluid Venereal Disease Research	This is a test that measures the amount of virus present. A positive test will show as "detected" or as a number. A negative or undetectable test will show as "not detected" or a number less than the lowest number in the range (for example: <200). You don't need to call us for a negative test. We'll let you know if there are any problems.

© 2025 Steitz BD et al. *JAMA Network Open*.

Test Result Category	Included Tests	Education Content
	Laboratory Titer (Syphilis Test)<br>• Immunohematology Reference Lab Isohemagglutinin Titer<br>• Antimitochondrial Antibody Screening<br>• Dipeptidyl-Peptidase-Like Protein 6 Antibody Indirect Fluorescent Antibody Titer<br>• Glial Fibrillary Acidic Protein Antibody Indirect Fluorescent Antibody Titer<br>• Metabotropic Glutamate Receptor 1 Antibody Indirect Fluorescent Antibody Titer<br>• Immunoglobulin-Like Cell Adhesion Molecule 5 Antibody Indirect Fluorescent Antibody Titer<br>• Neuronal Intermediate Filament Immunoglobulin G Indirect Fluorescent Antibody Titer<br>• Solid Organ Donor Preliminary Flow Cytometry Titer<br>• Solid Organ Recipient Preliminary Flow Cytometry Titer<br>• Cerebrospinal Fluid Dipeptidyl-Peptidase-Like Protein 6 Antibody Indirect Fluorescent Antibody Titer<br>• Cerebrospinal Fluid Glial Fibrillary Acidic Protein Antibody Indirect Fluorescent Antibody Titer<br>• Cerebrospinal Fluid Immunoglobulin-Like Cell Adhesion Molecule 5 Antibody Indirect Fluorescent Antibody Titer<br>• Cerebrospinal Fluid Metabotropic Glutamate Receptor 1 Antibody Indirect Fluorescent Antibody Titer<br>• Cerebrospinal Fluid Neuronal Intermediate Filament Immunoglobulin G Indirect Fluorescent Antibody Titer	

© 2025 Steitz BD et al. *JAMA Network Open*.

Test Result Category	Included Tests	Education Content
	• Antiglial Nuclear Antibody Type 1 Titer<br>• Amphiphysin Antibody Titer<br>• Antineuronal Nuclear Antibody Titer<br>• Collapsin Response-Mediator Protein 5 Immunoglobulin G Titer<br>• Purkinje Cell Cytoplasmic Antibody Titer<br>• Cerebrospinal Fluid Antiglial Nuclear Antibody Type 1 Titer<br>• Cerebrospinal Fluid Amphiphysin Antibody Titer<br>• Cerebrospinal Fluid Antineuronal Nuclear Antibody Titer<br>• Cerebrospinal Fluid Collapsin Response-Mediator Protein 5 Immunoglobulin G Titer<br>• Cerebrospinal Fluid Purkinje Cell Cytoplasmic Antibody Titer<br>• Neurochondrin Antibody Indirect Fluorescent Antibody Titer<br>• Septin-7 Antibody Indirect Fluorescent Antibody Titer<br>• (1,3)-Beta-D-Glucan Assay (Fungitell) Titer<br>• Cerebrospinal Fluid (1,3)-Beta-D-Glucan Assay (Fungitell) Titer<br>• Bronchoalveolar Lavage (1,3)-Beta-D-Glucan Assay (Fungitell) Titer<br>• Aquaporin-4 Immunoglobulin G Antibody with Reflex Titer<br>• Aquaporin-4 Immunoglobulin G Antibody Titer<br>• Cerebrospinal Fluid Phosphodiesterase 10A Antibody Indirect Fluorescent Antibody Titer<br>• Cerebrospinal Fluid Tripartite Motif-Containing 46 Antibody Cell-Based Assay	

© 2025 Steitz BD et al. *JAMA Network Open*.

Test Result Category	Included Tests	Education Content
	• Cerebrospinal Fluid Tripartite Motif-Containing 46 Antibody Indirect Fluorescent Antibody Titer<br>• Phosphodiesterase 10A Antibody Indirect Fluorescent Antibody Titer<br>• Tripartite Motif-Containing 46 Antibody Cell-Based Assay<br>• Tripartite Motif-Containing 46 Antibody Indirect Fluorescent Antibody Titer<br>• Endomysial Immunoglobulin A by Indirect Fluorescent Antibody<br>• Celiac Disease Panel with Reflex to Endomysial Antibody	

© 2025 Steitz BD et al. *JAMA Network Open*.

eTable 2. Characteristics of Patients Who Reviewed Test Results, Stratified by Test Type

Characteristic	Test Results, No. (%)							*P* value
	**BMP**	**CMP and LFTs**	**CBC**	**Microbial, Antibody, & PCR**	**Thyroid Panel and TSH**	**Urine Microalbumin**	**Total**	
	(N= 59,229)	(N=314,242)	(N=284,854)	(N= 20,686)	(N= 30,611)	(N=120,280)	(N=829,902)	
Age Group								<.001
18-34	6,867 (11.6)	62,369 (19.8)	45,723 (16.1)	2,019 (9.8)	5,986 (19.6)	29,508 (24.5)	152,472 (18.4)	
35-49	10,691 (18.1)	68,039 (21.7)	62,229 (21.8)	3,532 (17.1)	7,594 (24.8)	29,730 (24.7)	181,815 (21.9)	
50-64	17,154 (29.0)	89,573 (28.5)	87,931 (30.9)	6,941 (33.6)	10,769 (35.2)	31,069 (25.8)	243,437 (29.3)	
65-84	22,675 (38.3)	89,652 (28.5)	84,988 (29.8)	7,867 (38.0)	6,236 (20.4)	28,281 (23.5)	239,699 (28.9)	
85+	1,842 (3.1)	4,609 (1.5)	3,983 (1.4)	327 (1.6)	26 (0.1)	1,692 (1.4)	12,479 (1.5)	
Sex								<.001
Female	32,305 (54.5)	189,323 (60.2)	164,942 (57.9)	10,538 (50.9)	12,535 (40.9)	86,344 (71.8)	495,987 (59.8)	
Male	26,924 (45.5)	124,914 (39.8)	119,909 (42.1)	10,148 (49.1)	18,076 (59.1)	33,936 (28.2)	333,907 (40.2)	
Unknown	0 (0)	5 (0.0)	3 (0.0)	0 (0)	0 (0)	0 (0)	8 (0.0)	
Ethnicity								<.001
Hispanic or Latino	2,248 (3.8)	15,160 (4.8)	12,340 (4.3)	841 (4.1)	1,839 (6.0)	6,040 (5.0)	38,468 (4.6)	
Not Hispanic or Latino	51,997 (87.8)	272,216 (86.6)	246,842 (86.7)	17,668 (85.4)	26,999 (88.2)	102,143 (84.9)	717,865 (86.5)	
Other or unknown	4,984 (8.4)	26,866 (8.5)	25,672 (9.0)	2,177 (10.5)	1,773 (5.8)	12,097 (10.1)	73,569 (8.9)	
Race								<.001
American Indian or Alaska Native	255 (0.4)	1,393 (0.4)	1,247 (0.4)	91 (0.4)	178 (0.6)	482 (0.4)	3,646 (0.4)	
Asian	1,245 (2.1)	7,440 (2.4)	6,628 (2.3)	585 (2.8)	746 (2.4)	3,499 (2.9)	20,143 (2.4)	
Black or African American	7,849 (13.3)	33,396 (10.6)	28,451 (10.0)	3,221 (15.6)	6,135 (20.0)	10,174 (8.5)	89,226 (10.8)	
Middle Eastern or North African	178 (0.3)	1,310 (0.4)	1,029 (0.4)	70 (0.3)	124 (0.4)	568 (0.5)	3,279 (0.4)	
Native Hawaiian or Pacific Islander	96 (0.2)	403 (0.1)	290 (0.1)	21 (0.1)	101 (0.3)	139 (0.1)	1,050 (0.1)	
White	46,856 (79.1)	252,712 (80.4)	232,561 (81.6)	15,668 (75.7)	21,692 (70.9)	97,480 (81.0)	666,969 (80.4)	
Other or unknown	2,750 (4.6)	17,588 (5.6)	14,648 (5.1)	1,030 (5.0)	1,635 (5.3)	7,938 (6.6)	45,589 (5.5)	
Preferred Language								<.001
English	58,438 (98.7)	308,688 (98.2)	281,052 (98.7)	20,362 (98.4)	29,887 (97.6)	118,281 (98.3)	816,708 (98.4)	
Spanish	445 (0.8)	2,442 (0.8)	1,834 (0.6)	190 (0.9)	266 (0.9)	967 (0.8)	6,144 (0.7)	
Other	346 (0.6)	3,112 (1.0)	1,968 (0.7)	134 (0.6)	458 (1.5)	1,032 (0.9)	7,050 (0.8)	
Insurance								<.001
Commercial	29,647 (50.1)	185,179 (58.9)	170,809 (60.0)	11,614 (56.1)	16,632 (54.3)	81,076 (67.4)	494,957 (59.6)	
Medicaid	1,159 (2.0)	12,717 (4.0)	7,114 (2.5)	287 (1.4)	1,602 (5.2)	3,992 (3.3)	26,871 (3.2)	
Medicare	26,478 (44.7)	103,532 (32.9)	96,881 (34.0)	8,279 (40.0)	11,027 (36.0)	30,500 (25.4)	276,697 (33.3)	
Uninsured	840 (1.4)	4,037 (1.3)	3,519 (1.2)	215 (1.0)	344 (1.1)	1,517 (1.3)	10,472 (1.3)	
Other	1,105 (1.9)	8,777 (2.8)	6,531 (2.3)	291 (1.4)	1,006 (3.3)	3,195 (2.7)	20,905 (2.5)	
Years Enrolled in Portal								<.001
Mean (SD)	3.6 (2.4)	3.3 (2.4)	3.4 (2.4)	4.0 (2.3)	3.1 (2.4)	3.4 (2.4)	3.4 (2.4)	
Median (IQR)	3.7 [1.4, 6.1]	3.3 [1.1, 5.9]	3.4 [1.2, 6.0]	4.4 [2.2, 6.2]	2.9 [1.0, 5.7]	3.4 [1.3, 5.9]	3.4 [1.2, 6.0]	
Order Specialty								<.001
Primary Care	6,740 (11.4)	53,680 (17.1)	55,431 (19.5)	6,436 (31.1)	719 (2.3)	38,102 (31.7)	161,108 (19.4)	
Other	52,489 (88.6)	260,562 (82.9)	229,423 (80.5)	14,250 (68.9)	29,892 (97.7)	82,178 (68.3)	668,794 (80.6)	
Patient First Review								<.001
No	18,477 (31.2)	93,785 (29.8)	90,428 (31.7)	5,965 (28.8)	11,139 (36.4)	34,526 (28.7)	254,320 (30.6)	
Yes	40,752 (68.8)	220,457 (70.2)	194,426 (68.3)	14,721 (71.2)	19,472 (63.6)	85,754 (71.3)	575,582 (69.4)	
Test Findings								
Abnormal	12,436 (21.0)	81,443 (25.9)	63,475 (22.3)	9,285 (44.9)	29,337 (95.8)	102,103 (84.9)	298,079 (35.9)	
Normal	46,793 (79.0)	232,799 (74.1)	221,379 (77.7)	11,401 (55.1)	1,274 (4.2)	18,177 (15.1)	531,823 (64.1)	
Patient-initiated message within 24 hours								<.001
No	47,551 (80.3)	259,417 (82.6)	235,735 (82.8)	17,682 (85.5)	22,609 (73.9)	102,285 (85.0)	685,279 (82.6)	
Yes	11,678 (19.7)	54,825 (17.4)	49,119 (17.2)	3,004 (14.5)	8,002 (26.1)	17,995 (15.0)	144,623 (17.4)	

© 2025 Steitz BD et al. *JAMA Network Open*.
