# Supplementary material for: Patient-Friendly Test Results and Patient-Initiated Messaging Among Adult Outpatients
Source: JAMA Netw Open. 2025 Nov 17;8(11):e2543879. doi: 10.1001/jamanetworkopen.2025.43879 (PMC12625389; doi:10.1001/jamanetworkopen.2025.43879)
Supplement: Supplement 2. — Data Sharing Statement [file jamanetwopen-e2543879-s002.pdf]

Data Sharing Statement

Steitz. Patient-Friendly Test Results and Patient-Initiated Messaging Among Adult Outpatients. *JAMA Netw Open*. Published November 17, 2025. doi:10.1001/jamanetworkopen.2025.43879

Data
Data available: No

Additional Information
Explanation for why data not available: The study dataset contains record-level detail of patients and clinicians, which we are not allowed to share.
